# High-fat diet impact on intestinal cholesterol conversion by the microbiota and serum cholesterol levels

**DOI:** 10.1016/j.isci.2023.107697

**Published:** 2023-08-21

**Authors:** Alena M. Bubeck, Paul Urbain, Cathrine Horn, Anna S. Jung, Lisa Ferrari, Hannah K. Ruple, Daniel Podlesny, Stefanie Zorn, Johnny Laupsa-Borge, Caroline Jensen, Inge Lindseth, Gülen Arslan Lied, Jutta Dierkes, Gunnar Mellgren, Hartmut Bertz, Silke Matysik, Sabrina Krautbauer, Gerhard Liebisch, Hans-Frieder Schoett, Simon N. Dankel, W. Florian Fricke

**Affiliations:** 1 Department of Microbiome Research and Applied Bioinformatics, Institute of Nutritional Sciences, University of Hohenheim, Stuttgart, Germany; 2 Department of Medicine I, Medical Center – University of Freiburg, Faculty of Medicine, University of Freiburg, Freiburg, Germany; 3 Mohn Nutrition Research Laboratory, Centre for Nutrition, Department of Clinical Science, University of Bergen, Bergen, Norway; 4 Mohn Nutrition Research Laboratory, Centre for Nutrition, Department of Clinical Medicine, University of Bergen, Bergen, Norway; 5 Balderklinikken, Oslo, Norway; 6 Department of Hematology, Oncology and Stem Cell Transplantation, Medical Center - University of Freiburg, Faculty of Medicine, University of Freiburg, Freiburg, Germany; 7 Institute of Clinical Chemistry and Laboratory Medicine, University Hospital Regensburg, Regensburg, Germany; 8 Singapore Lipidomics Incubator (SLING), Life Sciences Institute, National University of Singapore, Singapore, Singapore; 9 Institute for Genome Sciences, University of Maryland School of Medicine, Baltimore, MD, USA

**Keywords:** Health sciences, Human metabolism, Microbiology, Genomics

## Abstract

Cholesterol-to-coprostanol conversion by the intestinal microbiota has been suggested to reduce intestinal and serum cholesterol availability, but the relationship between intestinal cholesterol conversion and the gut microbiota, dietary habits, and serum lipids has not been characterized in detail. We measured conserved proportions of cholesterol high and low-converter types in individuals with and without obesity from two distinct, independent low-carbohydrate high-fat (LCHF) dietary intervention studies. Across both cohorts, cholesterol conversion increased in previous low-converters after LCHF diet and was positively correlated with the fecal relative abundance of *Eubacterium coprostanoligenes*. Lean cholesterol high-converters had increased serum triacylglycerides and decreased HDL-C levels before LCHF diet and responded to the intervention with increased LDL-C, independently of fat, cholesterol, and saturated fatty acid intake. Our findings identify the cholesterol high-converter type as a microbiome marker, which in metabolically healthy lean individuals is associated with increased LDL-C in response to LCHF.

## Highlights

• Equal fractions of cholesterol high/low-converters in humans with or without obesity
• Increased *E. coprostanol.* in high-converters but no reduction in serum cholesterol
• Diet increased conversion in low-converters without altering serum cholesterol
• Increased TAG before and LDL-C after low-carb high-fat diet in lean high-converters

## Graphical abstract

**Figure:**
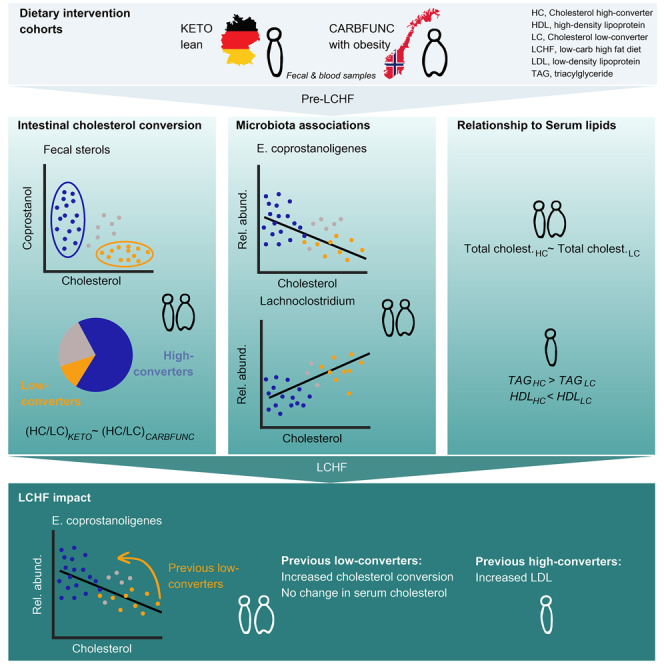

## Introduction

Cholesterol, an amphipathic sterol lipid, is an essential structural component of human and animal cell membranes and serves as a precursor for the biosynthesis of steroid hormones, bile acids and vitamin D. However, hypercholesterolemia, or excess blood cholesterol, is a major risk factor for cardiovascular disease, the leading cause of mortality worldwide.^1^ Large amounts of cholesterol enter the small intestine every day from exogenous dietary sources, including animal products (∼0.3–0.6 g/day), and from endogenous biosynthesis in the liver and secretion with bile acids (∼0.7–0.9 g/day).^2^ Consequently, hypercholesterolemia treatment can involve limiting dietary cholesterol intake, inhibiting cholesterol biosynthesis, and/or blocking cholesterol uptake from the intestine. Statins, pharmacological inhibitors of the HmG-CoA-dependent cholesterol-generating mevalonate pathway in the liver, represent one of the most successful, widely used and best-selling drug classes worldwide.^3^ Lowering dietary cholesterol intake alone typically has limited and inconsistent effects,^4^ as cholesterol production is tightly controlled via feedback mechanisms that adapt HmG-CoA activity to dietary cholesterol intake and cellular requirements.^5^ These mechanisms involve insulin, which can increase or decrease HmG-CoA expression in response to dietary carbohydrates^6^ and low-carbohydrate diets can reduce circulating cholesterol levels.^7^ The clinical importance of cholesterol availability in the intestine is demonstrated by the successful combination of statins with small-molecule inhibitors of cholesterol uptake^8^ and may be influenced by the gut microbiota.^9^ Intestinal bacteria can metabolize statins^10^ and may contribute to inconsistent and adverse effects of the medication in some individuals.^11^^,^^12^ Thus, a better understanding of the relationship between intestinal cholesterol availability and circulating cholesterol levels in relation to the gut microbiota could identify diagnostic markers for patient stratification, as well as therapeutic targets for the development of new cholesterol-reducing interventions.

The intestinal microbiota can reduce cholesterol to coprostanol, which is unavailable for human absorption, stable under anoxic conditions and excreted in feces.^13^ Fecal coprostanol is reduced in antibiotically treated animals and humans^14^^,^^15^ and undetectable in germ-free rats^16^ and human newborns.^17^ Intestinal cholesterol conversion has been attributed to a broad and diverse range of microbial taxa, based on *in vitro* experiments with bacterial isolates from feces and *in silico* fecal metagenomic sequence analysis,^18^ but few human or animal fecal bacterial isolates with confirmed cholesterol conversion activity are available.^2^ The rate of cholesterol-to-coprostanol conversion varies between individuals but, based on the fecal coprostanol/cholesterol ratio, shows a stable bimodal distribution into cholesterol high and low-converters in healthy human populations.^19^^,^^20^^,^^21^ Yet, whether intestinal cholesterol conversion by the human gut microbiota remains stable over a person’s lifetime, to what extent it is affected by dietary changes or other endogenous or exogenous factors, or whether it affects cholesterol availability to the human host remains unclear and has been controversially discussed.^2^

Low-carbohydrate high-fat (LCHF) diets have become increasingly popular in recent years to induce weight loss and improve blood sugar control in individuals with obesity.^6^^,^^22^ Ketogenic LCHF diets aim to induce a shift of the body’s primary dietary energy source from carbohydrates to fats, resulting in a state of ketosis and far-reaching physiological alterations.^23^ Besides obesity reduction, LCHF diets have been associated with reduced cardiovascular disease risks, improved type 2 diabetes, and other health benefits^24^ and, as a consequence, become popular among young, healthy, normal-weight people.^25^ However, LCHF diets have also been reported to increase serum concentrations of low-density lipoprotein cholesterol (LDL-C), a risk marker for atherosclerotic cardiovascular disease, in a subset of individuals, including metabolically healthy, normal-weight adults.^26^^,^^27^^,^^28^^,^^29^ Although the clinical relevance of this elevated LDL-C response to LCHF diets in individuals without insulin resistance has been debated,^30^ as well as the benefits of statin therapy to treat it,^31^ there is a need to identify affected individuals and avert potential negative consequences of these popular diets, especially in healthy young populations.

In this study, we examine the relationship between intestinal cholesterol-to-coprostanol conversion, gut microbiota, and serum lipid profiles, in the context of LCHF interventions. We identify the stratification of individuals into cholesterol high and low-converter types as a conserved feature of the fecal microbiome in two human cohorts with distinct geographic (Germany/Norway) and metabolic (with/without obesity) study parameters. Across both cohorts, we find cholesterol conversion to be strongly correlated to the fecal relative abundance of *E. coprostanoligenes* and to be increased in previous low-cholesterol converters on (ketogenic/non-ketogenic) LCHF diets without affecting circulating cholesterol levels. Finally, we report the cholesterol high-converter type to be associated with adverse circulating lipid profiles in metabolically healthy lean individuals (increased triacylglycerides [TAG] and decreased high-density lipoprotein cholesterol [HDL-C]), who also respond to ketogenic LCHF with increased LDL-C levels. Thus, while the cholesterol high-converter type may not be associated with reduced serum cholesterol levels, it may be indicative in metabolically healthy lean individuals of adverse blood lipid profiles and increased LDL-C responses to LCHF diets.

## Results

### Equal distributions of cholesterol high and low-converter types among humans with and without obesity

**Figure 1:**
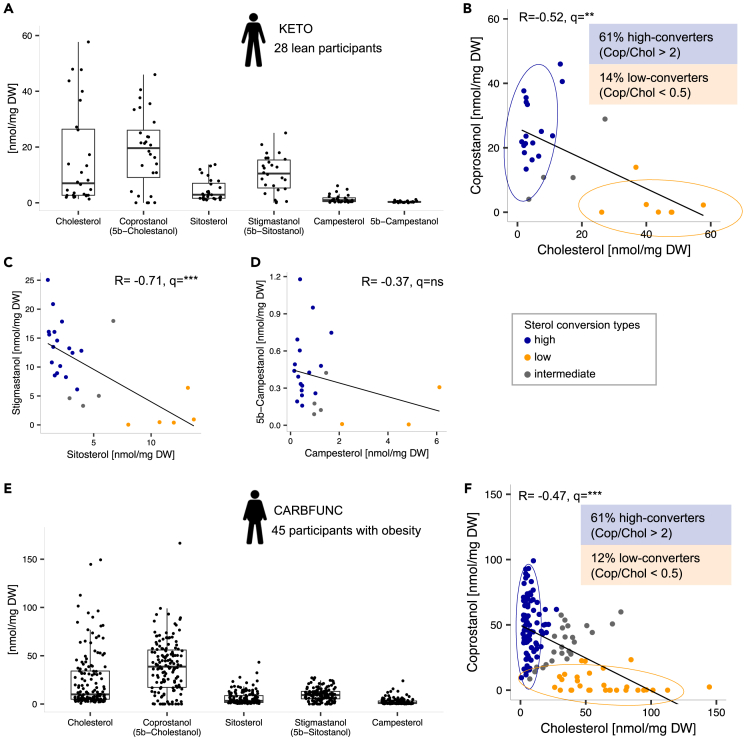
Equal distributions of cholesterol high and low-converter types among humans with and without obesity (A) Sterol and stanol concentrations as determined by LC-MS/HRMS in 28 fecal samples from the KETO study participants before dietary intervention. (B) Negative correlation (Spearman’s rank) between fecal coprostanol and cholesterol concentrations and bimodal distribution of cholesterol high (N = 17), intermediate (N = 4) and low-converter (N = 7) types, as classified based on the fecal coprostanol/cholesterol ratio. (C and D) Comparable negative correlations (Spearman’s rank) between the fecal concentrations of the phytosterols sitosterol and campesterol and the corresponding stanol conversion products stigmastanol (n = 26), and 5β-campestanol (n = 23). (E) Similar fecal sterol and stanol concentration profiles in individuals with obesity from the CARBFUNC study before dietary intervention (n = 145 samples), compared to lean KETO study participants (N = 89/26/30 for cholesterol high/intermediate/low-converters). (F) Negative correlation (Spearman’s rank) of fecal coprostanol and cholesterol concentrations in CARBFUNC study participants and bimodal distribution into high and low-converter types. Spearman’s rank correlation, Benjamini-Hochberg (BH) corrected: q > 0.05 ns, q < 0.01 ∗∗, q < 0.001 ∗∗∗. Pooled data are represented as mean ± SD.

Fecal sterol and stanol concentrations were determined by liquid chromatography-high resolution mass spectrometry (LC-MS/HRMS) in 28 healthy, normal-weight participants of a German ketogenic (KETO) diet study (German Clinical Trials Register: DRKS00009605,^32^). Before dietary intervention, the microbial conversion products coprostanol (18.71 ± 13.44 nmol/mg dry weight [DW]) and stigmastanol (5β-sitostanol) (10.39 ± 6.71 nmol/mg DW) were the most abundant fecal stanols, followed by their animal and plant-derived sterol precursors, fecal cholesterol (15.76 ± 17.53 nmol/mg DW), β-sitosterol (4.62 ± 3.99 nmol/mg DW) and campesterol (1.43 ± 1.51 nmol/mg DW) (Figure 1A). All sterol and stanol concentrations exhibited substantial interindividual variation and, as would be expected from a direct metabolic dependency, coprostanol and cholesterol levels were negatively correlated (Figure 1B, R = −0.52, q = 0.005, Spearman’s rank correlation, corrected for false discovery rate [FDR]), similarly to stigmastanol and β-sitosterol levels (Figure 1C, R = −0.71, q < 1e-5) and 5β-campestanol and campesterol levels (Figure 1D, R = −0.37, q = 0.073). The KETO study participants showed a bimodal distribution into high and low cholesterol-to-coprostanol converter types, as previously described for healthy individuals,^19^^,^^20^ i.e., a greater fraction of high (coprostanol/cholesterol ratio > 2, 61% of the KETO study participants) compared to low (coprostanol/cholesterol ratio < 0.5, 14% of the KETO study participants) cholesterol converters (Figure 1B). The remainder (25% of the KETO study participants) was classified as intermediate cholesterol converters.

To compare cholesterol high and low-converter type fractions among individuals from independent cohorts and with different metabolic health backgrounds, fecal sterol and stanol concentrations were also characterized in 145 individuals with obesity from the CARBFUNC study, a Norwegian 2-year randomized controlled dietary intervention trial (ClinicalTrials.gov: NCT03401970). The main characteristics of both study cohorts are listed in Table S1. Individuals with obesity from the CARBFUNC study exhibited increased concentrations of fecal coprostanol (Table S1, p < 1e-3, Wilcoxon rank-sum test), but otherwise similar sterol or stanol levels (p > 0.05) and compositional profiles (Figure 1E), including a negative correlation of coprostanol to cholesterol (R = −0.47, q < 1e-9) and a bimodal distribution into cholesterol high (61% of CARBFUNC study participants) and low (21% of CARBFUNC study participants) converter types based on the fecal coprostanol/cholesterol ratios (Figure 1F). Stratification of individuals into larger cholesterol high-converter and smaller cholesterol low-converter type fractions therefore appears to be a conserved feature of the human fecal microbiome in individuals with and without obesity.

### Distinct microbiota associations with fecal cholesterol and coprostanol

To better understand the intra-intestinal relationship of cholesterol-to-coprostanol conversion with the gut microbiota, fecal taxonomic microbiota, and metabolite profiles were compared between cholesterol high and low-converters from the KETO and CARBFUNC studies. Fecal samples from both cohorts were independently analyzed using different 16S rRNA gene amplicon sequencing protocols, presenting a potential confounding factor for the direct comparison of taxonomic microbiota profiles from both cohorts but also allowing for the identification of robust associations with cholesterol conversion.

**Figure 2:**
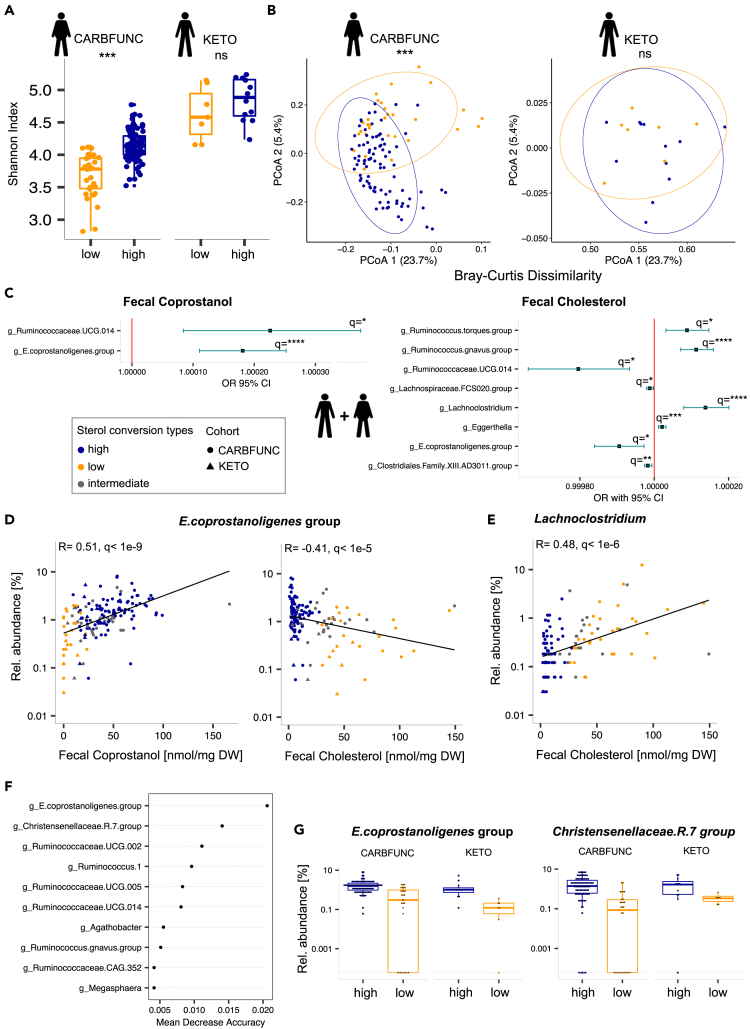
Distinct microbiota associations with fecal cholesterol and coprostanol (A and B) Reduced diversity (Wilcoxon rank-sum) and altered composition (ANOSIM) of taxonomic microbiota profiles of cholesterol high (N = 89) compared to low-converters (N = 30) with obesity from the CARBFUNC study, but no difference between lean cholesterol high (N = 7) and low-converters (N = 12) from the KETO study. p > 0.05 ns, p < 0.001 ∗∗∗. (C) Positive and negative associations of bacterial taxa with fecal coprostanol and cholesterol concentrations, as identified by a generalized linear mixed model (GLMM) for the combined dataset of KETO (N = 23) and CARBFUNC (N = 145) study participants. For the GLMM input, zero values were replaced with a pseudocount and cohort and gender added as random and fixed effects (see STAR Methods for details). (D and E) Across both cohorts combined, *Eubacterium coprostanoligenes*.group (N = 158) was positively correlated with fecal coprostanol and negatively correlated with fecal cholesterol concentrations, whereas *Lachnoclostridium* (N = 125), was positively correlated with fecal cholesterol concentrations. (F) *E. coprostanoligenes.*group relative abundance was the most informative microbiota feature for predicting the cholesterol converter type with a random forest model, based on leave-one-out cross-validation (LOOCV). (G) Increased relative abundance of *E. coprostanoligenes*.group and *Christensenellaceae*.R.7.group in cholesterol high-converters. Dashed lines indicate pseudocount values (0.0001% relative abundance) of samples with zero taxon counts. Benjamini-Hochberg (BH) corrected: q > 0.1 ns, q < 0.1 ∗, q < 0.05 ∗∗, q < 0.01 ∗∗∗, q < 0.001 ∗∗∗∗. Pooled data are represented as mean ± SD.

No difference in fecal microbiota ɑ-diversity (Figure 2A, Shannon index, p = 0.17, Wilcoxon rank-sum test) or β-diversity (Figure 2B, Bray-Curtis dissimilarity, R = 0.084, p = 0.15, ANOSIM) was detected between lean cholesterol high and low-converters from the KETO study. However, low-converters with obesity from the CARBFUNC study had a reduced fecal microbiota ɑ-diversity (Figure 2A, Shannon, p < 1e-9, Wilcoxon rank-sum test) and distinct β-diversity (Figure 2B, Bray-Curtis dissimilarity, R = 0.24, p = 0.001, ANOSIM) relative to high-converters, suggesting that the microbiota relationship to cholesterol conversion was affected by obesity or other CARBFUNC study-specific factors.

Next, a generalized linear mixed model (GLMM) was used to identify shared linear associations between specific members of the fecal microbiota and fecal cholesterol and coprostanol concentrations across both the KETO and CARBFUNC studies combined (Table S2). Whereas coprostanol detected in feces should originate exclusively from microbial cholesterol reduction, fecal cholesterol could have both endogenous and exogenous origins.^2^ Fecal coprostanol and cholesterol concentrations were therefore queried for associations with the centered log-ratio (clr)-transformed relative abundances of specific bacterial taxa both independently and in combination, while controlling in the model for cohort and gender-specific effects (Figures 2C and S1, and Table S2). Significant associations from the GLMMs (q < 0.1) were independently assessed by Spearman’s rank correlation analysis (Figures 2D, 2E, and S1). Only two bacterial taxa, i.e., *E. coprostanoligenes*.group and *Ruminococcaceae*.UCG.014, showed consistent associations with cholesterol-to-coprostanol conversion across all comparisons and analyses, i.e., positive and negative correlations to fecal coprostanol and cholesterol levels, respectively (Figures 2C, 2D, and S1, Spearman’s rank correlation^FDR^), and a positive association with the coprostanol/cholesterol ratio (Figure S1). In addition, *Lachnoclostridium* was positively and *Clostridiales*.XIII.AD3011 negatively correlated to fecal cholesterol concentrations (Figures 2E and S1, Spearman’s rank correlation^FDR^). Detection of *Clostridiales.*XIII.AD3011 was limited to the CARBFUNC cohort (Figure S1), indicating obesity, technical or otherwise study-related link of this genus to fecal cholesterol.

To also test for non-linear microbiota associations with the cholesterol high and low converter types, a random forest classifier was trained on clr-transformed microbiota compositions. This classifier performed well at identifying cholesterol high-converters (84.82% precision, 94.06% recall), but lacked sensitivity for the detection of low-converters (76.92% precision, 54.05% recall). In line with the GLMM, leave-one-out cross-validation (LOOCV) identified *E. coprostanoligenes*.group as the most important bacterial taxon for this classification, whereas several others detected by LOOCV, such as *Christensenellaceae*.R.7.group, were only correlated to fecal cholesterol levels by the GLMM and flagged with singularity fit warnings, indicating potential overfit of the linear model (Figures 2F and 2G). In summary, linear and non-linear models consistently linked *E. coprostanoligenes* to intestinal cholesterol-to-coprostanol conversion across both cohorts. However, other bacterial taxa may also be involved in the process.

**Figure 3:**
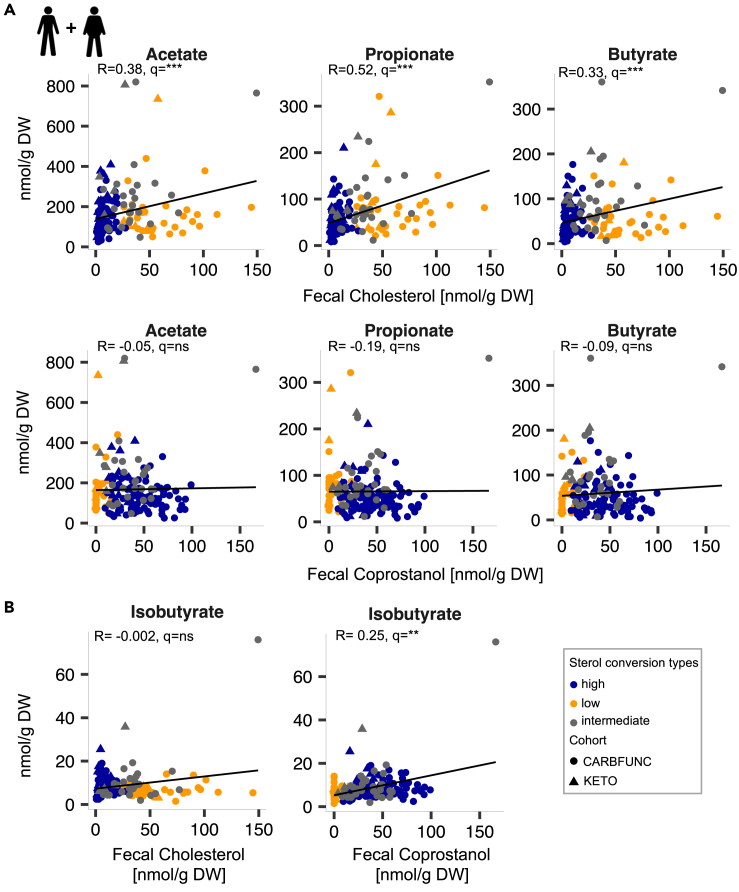
Distinct associations of fecal cholesterol and coprostanol concentrations with short and branched-chain fatty acids (A and B) Positive correlation of fecal cholesterol with the concentrations of the SCFAs acetate, propionate and butyrate (A) and of fecal coprostanol with the BCFA isobutyrate (B) in fecal samples from lean KETO study participants (N = 28) and in individuals with obesity from the CARBFUNC study (N = 145) before the dietary intervention. Spearman’s rank correlation, BH-corrected: q > 0.05 ns, q < 0.05 ∗, q < 0.01 ∗∗, q < 0.001 ∗∗∗.

Next, fecal cholesterol and coprostanol levels were compared to short-chain fatty acid (SCFAs) and branched-chain fatty acid (BCFAs) concentrations in stool samples. SCFAs are mainly produced by microbial fermentation of non-digestible dietary fiber in the colon, whereas BCFAs predominantly result from microbial protein fermentation (Wolter et al.^33^). Fecal cholesterol but not coprostanol was positively correlated across both studies to the SCFAs acetate (R = 0.38, q < 1e-4), propionate (R = 0.52, q < 1e-9) and butyrate (R = 0.33, q < 1e-3) (Figure 3A, Spearman’s rank correlation^FDR^). In contrast, fecal coprostanol but not cholesterol, showed a positive correlation to the BCFA isobutyrate (Figure 3B, R = 0.25, q = 0.005, Spearman’s rank correlation^FDR^). An association of the cholesterol converter type with fecal SCFA or BCFA levels was only identified in study participants with obesity (CARBFUNC), including decreased fecal propionate (p < 1e-4) and increased isobutyrate (p = 0.023) concentrations in high-converters (Wilcoxon rank-sum test). Thus, our findings are in agreement with previous reports of increased SCFA secretion in cholesterol low-converters.^34^ They indicate distinct intra-intestinal associations of cholesterol and coprostanol with specific microbial taxa and metabolites, which may be influenced by obesity or other cohort-specific parameters.

### Circulating blood lipids in cholesterol high and low-converters

**Figure 4:**
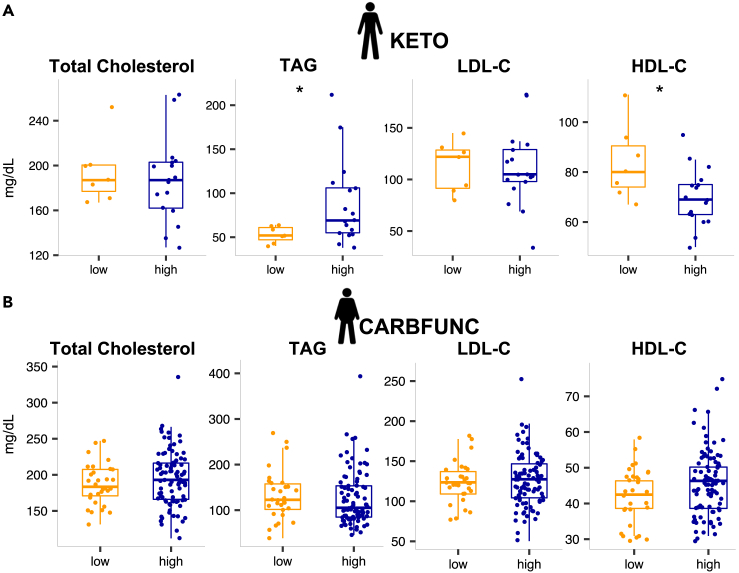
Circulating blood lipids in cholesterol high and low-converters (A) Comparable total cholesterol, but increased serum TAG and decreased HDL-C levels in lean cholesterol low (N = 7) compared to high-converters (N = 17) from the KETO study. (B) No significant difference in blood lipid levels between cholesterol high (N = 89) and low-converters (N = 30) with obesity from the CARBFUNC study. Wilcoxon rank-sum, p > 0.05 ns, p < 0.05 ∗. Pooled data are represented as mean ± SD.

To determine if intestinal cholesterol-to-coprostanol conversion types were reflected in altered circulating cholesterol levels, we compared fecal cholesterol and coprostanol concentrations with the serum levels of total cholesterol and other lipids. Lean cholesterol high and low-converters showed comparable total cholesterol and LDL-C concentrations before the intervention (Figure 4A, KETO cohort, p > 0.05, Wilcoxon rank-sum test), but TAG levels were increased (p = 0.04) and HDL-C levels decreased (p = 0.04) in lean cholesterol high-converters (Figure 4A, Wilcoxon rank-sum test). Cholesterol high and low-converters with obesity showed no difference in serum total cholesterol, TAG, HDL-C, and LDL-C (Figure 4B, CARBFUNC cohort, p > 0.05, Wilcoxon rank-sum test). However, CARBFUNC study participants had increased serum TAG (p < 1e-7), LDL-C (p = 0.03) and reduced serum HDL-C (p < 1e-13) levels compared to KETO study participants and showed an increased TAG/HDL-C ratio (p < 1e-11), a marker for insulin resistance,^35^ consistent with generally adverse health profiles in the cohort with obesity (Table S1). In line with this notion, CARBFUNC study participants had increased blood glucose and insulin levels (Table S1), but no difference was observed for either of these parameters between cholesterol high and low-converters from both studies (KETO: blood glucose p = 0.092, insulin p = 0.77; CARBFUNC: blood glucose p = 0.66, insulin p = 0.14, Wilcoxon rank-sum test), and neither glucose nor insulin were significantly correlated with fecal coprostanol or cholesterol (q > 0.05, Spearman’s rank correlation^FDR^, Figure S2). However, compared to cholesterol low-converters, high-converters from the CARBFUNC study had elevated serum β-hydroxybutyric acid (BHB) levels (low: 39.2 ± 33.8 vs. high: 61.6 ± 59.2, p = 0.013, mean ± sd, Wilcoxon rank-sum Test), indicative of increased ketosis in the cholesterol high-converters with obesity.

Neither fecal coprostanol nor cholesterol levels were significantly correlated with serum total cholesterol, TAG, LDL-C, or HDL-C in either the CARBFUNC or KETO cohorts, or individuals from both studies combined (Figure S2, q > 0.05, Spearman’s rank correlation^FDR^). Similarly, no significant associations between fecal microbial taxa and serum lipid levels were identified by the GLMM after false discovery rate correction (Table S2, q > 0.1). Our findings therefore provide no indication for a link between increased intestinal cholesterol-to-coprostanol conversion and reduced circulating cholesterol levels.

### Diet impact on cholesterol-to-coprostanol conversion

**Figure 5:**
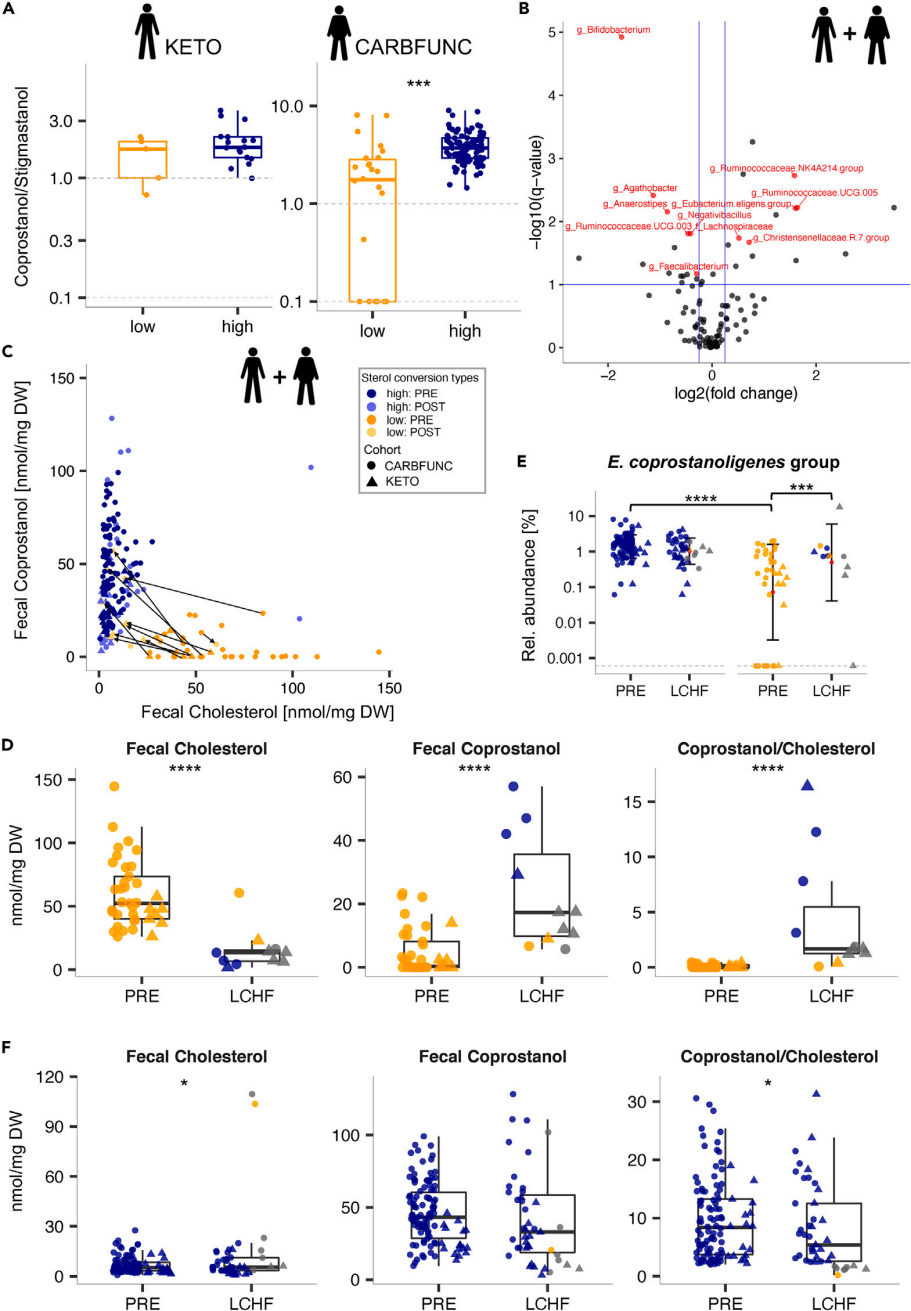
Diet impact on cholesterol-to-coprostanol conversion (A) Cholesterol high-converters with obesity (CARBFUNC study, N = 89) but not without obesity (KETO study, N = 17) exhibited an increased fecal coprostanol/stigmastanol ratio compared to low-converters (N_CARBFUNC_ = 26, N_KETO_ = 5), suggesting a higher proportion of animal vs. plant-derived dietary fat intake (Wilcoxon rank-sum, p < 0.001 ∗∗∗). Zero values were replaced with a pseudocount (1 nmol/mg dry weight [DW]). (B) LCHF diets induced consistent taxonomic microbiota alterations in the KETO and CARBFUNC cohorts, based on a combined GLMM analysis (N_PRE_ = 173, N_LCHF_ = 62). Bacterial taxa with significant changes in relative abundance (q < 0.1, BH-corrected Tukey’s test, see horizontal blue line) and a positive or negative fold-change of > 0.25 in estimated marginal means (EMM) are marked with red dots and labels, unless they were detected by the GLMM as cohort and/or sex-associated (black dots). (C) Distribution of cholesterol high and low-converters among all KETO and CARBFUNC study participants before (N = 143) and after (N = 54) LCHF dietary intervention (gray lines connecting pre and post-intervention samples). (D) Increased cholesterol-to-coprostanol conversion in low-converters from both cohorts on the LCHF diets (N_PRE_ = 37, N_LCHF_ = 11), as evidenced by reduced fecal cholesterol and increased fecal coprostanol levels and increased coprostanol/cholesterol ratios. (E) The increased cholesterol-to-coprostanol conversion in low-converters on LCHF diets was accompanied by an increased fecal relative abundance of *E. coprostanoligenes*.group (N_PRE, LOW_ = 37, N_LCHF, LOW_ = 11), resulting in similar relative abundances in high and low-converters on the LCHF diets (N_PRE, HIGH_ = 106, N_LCHF, HIGH_ = 43). (F) Decreased cholesterol-to-coprostanol conversion in high-converters from both cohorts after LCHF diet intervention (N_PRE_ = 106, N_LCHF_ = 43), at least based on increased fecal cholesterol levels and an increased coprostanol/cholesterol ratio. Individuals were classified as cholesterol high/low-converters based on pre-intervention time points, with symbol colors indicating the classification during the LCHF diet. Significance determined by GLMM and post-hoc Tukey’s test (Benjamini-Hochberg-corrected): q > 0.1 ns, q < 0.1 ∗, q < 0.05 ∗∗, q < 0.01 ∗∗∗, q < 0.001 ∗∗∗∗. Pooled data are represented as mean ± SD.

To identify associations between dietary habits and cholesterol-to-coprostanol conversion, lean KETO study participants were compared based on available food frequency questionnaire data. Caloric intake from fats, fiber, carbohydrates, protein, or cholesterol was comparable between lean cholesterol high and low-converters (Table 1, p > 0.05, Wilcoxon rank-sum test) and both converter types exhibited similar fecal fatty acid profiles, in terms of chain length and saturation level (Figure S3, p > 0.05, Wilcoxon rank-sum test). To estimate the ratio of animal to plant-derived fat intake, fecal coprostanol and stigmastanol levels were compared, as the former should originate mostly from conversion of cholesterol from animal origins (besides endogenous sources) and the latter mostly from conversion of the phytosterol β-sitosterol from plant origins.^2^ While no difference was detected between lean high and low-converters (Figure 5A, KETO cohort, p > 0.05, Wilcoxon rank-sum test), the fecal coprostanol/stigmastanol ratio was increased in cholesterol high-converters with obesity (Figure 5A, CARBFUNC cohort, p < 1e-5, Wilcoxon rank-sum test), indicating that, compared to low-converters, high-converters with obesity obtained a larger fraction of their fat intake from animal sources.

**Table 1:** Semi-quantitative food questionnaire-based dietary habits of lean KETO study participants

	PRE-LCHF			LCHF			PRE vs. LCHF	
	Low converter	High converter	Low vs. High	Low converter	High converter	Low vs. High	Low converter	High converter
	Mean ± SD	Mean ± SD	pPRE	Mean ± SD	Mean ± SD	pLCHF	p value	p value
Total kcal	2380.32 ± 394.11	2223.66 ± 454.11	0.43	2586.78 ± 835.78	2066.96 ± 425.61	0.14	0.43	0.24
Protein [%E]	13.56 ± 1.08	14.59 ± 2.21	0.35	19.83 ± 3.70	18.96 ± 3.55	0.81	0.03 (∗)	<1e-3 (∗∗∗)
Carbohydrate [%E]	42.16 ± 7.22	41.47 ± 6.15	0.92	5.81 ± 1.82	8.54 ± 3.09	0.06	0.03 (∗)	<1e-4 (∗∗∗)
Fat [%E]	40.13 ± 8.04	37.95 ± 5.30	0.66	72.15 ± 5.28	69.75 ± 5.85	0.56	0.03 (∗)	<1e-4 (∗∗∗)
SFA [%E]	17.17 ± 4.23	15.69 ± 2.40	0.52	26.71 ± 3.69	26.90 ± 3.96	0.92	0.03 (∗)	<1e-4 (∗∗∗)
MUFA [%E]	12.86 ± 3.54	12.74 ± 2.40	0.92	25.73 ± 3.35	26.20 ± 5.19	1.0	0.03 (∗)	<1e-4 (∗∗∗)
PUFA [%E]	5.09 ± 0.57	5.51 ± 1.62	0.61	9.54 ± 1.45	9.74 ± 2.99	0.81	0.03 (∗)	<1e-4 (∗∗∗)
Cholesterol [mg/d]	325.4 ± 165.9	304.5 ± 108.9	0.92	569.1 ± 141.5	429.8 ± 100.5	0.02 (∗)	0.03 (∗)	0.001 (∗∗)
Fiber [g/d]	24.6 ± 6.2	27.5 ± 14.1	0.97	26.5 ± 11.6	20.8 ± 7.1	0.29	0.84	0.02

Data were collected over seven consecutive days 1–2 weeks before (PRE) and during the last week of dietary intervention (LCHF) and used to calculate average daily intakes (+/− standard deviations [SD]) and determine significant differences between before and on LCHF time points (paired Wilcoxon signed-rank test) and between high/low-converter types (Wilcoxon rank-sum), N(low) = 6, N(high) = 17, p < 0.05 ∗, p < 0.01 ∗∗, p < 0.001 ∗∗∗, Abbreviations: SFA = saturated fats, MUFA = mono-unsaturated fats, PUFA = poly-unsaturated fats.

Both the KETO and CARBFUNC studies involved interventions with low-carbohydrate high-fat (LCHF) diets, based on ≥75 energy percent [E%] fat and ≤10 E% carbohydrate intake. Lean KETO study participants followed a 6-week *ad libitum* LCHF diet, which resulted in increased urinary and blood ketone bodies, as well as other hormonal and metabolic changes indicative of ketosis, as previously described elsewhere in detail.^32^^,^^36^ CARBFUNC study participants with obesity were restricted to a normocaloric (males: 2,500 kcal, females: 2,000 kcal) LCHF diet (https://clinicaltrials.gov/ct2/show/NCT03401970), which was accompanied by at least transient ketosis at three months of the intervention based on increased serum BHB levels (PRE: 62.68 μM +/− 68.67 vs. 3 months: 264.01 μM +/− 251.74, p = 0.00031; PRE vs. 6 months: 163.26 ± 274.60, p = 0.26; mean ± SD, paired Wilcoxon signed-rank test). Fecal and serum samples were collected after six weeks (KETO study) and three and six months (CARBFUNC study) and used for microbiota analysis and lipid profiling. As both studies included individuals with different metabolic health backgrounds and involved variable time spans, we first tested whether comparable taxonomic compositional microbiota alterations could be detected in both cohorts after LCHF diet intervention. A strong and consistent shift in microbiota compositions was detected across both cohorts (Figure 5B; Table S2, GLMM), including changes in the relative abundance of bacterial genera, such as *Bifidobacterium* (mean reduction: −4.23% +/− 2.48), previously reported to be altered by ketogenic diet.^37^ Our microbiota analysis therefore demonstrates reproducible, temporally stable LCHF diet-induced compositional microbiota changes in individuals with and without obesity.

To test whether intestinal cholesterol-to-coprostanol conversion could be dietarily modulated, we compared fecal cholesterol and coprostanol levels in KETO and CARBFUNC study participants in response to the LCHF diets using GLMMs (Figure 5C). These models identified cohort-specific effects of the LCHF diets on both fecal cholesterol and coprostanol concentrations but not their ratios (Figure 5D; Table S2). In cholesterol low-converters from both cohorts, LCHF diet increased the cholesterol-to-coprostanol conversion, as illustrated by decreased fecal cholesterol (q < 1e-7), increased fecal coprostanol (q < 1e-3) levels, and increased coprostanol/cholesterol (q < 1e-9) ratios (Figure 5D; Table S2, GLMM-based estimated marginal means [EMM]). The increased cholesterol conversion in low-converters on the LCHF diets was accompanied by a higher fecal relative abundance of *E. coprostanoligenes* (q = 0.003, Figure 5E; Table S2, GLMM-based EMMs). Cholesterol high-converters responded to the LCHF diets with reduced cholesterol conversion, at least based on increased fecal cholesterol (q = 0.08) concentrations and a decreased coprostanol/cholesterol ratio (q = 0.03), although fecal coprostanol levels were not altered (q > 0.1) (Figure 5F; Table S2, GLMM-based EMMs). No difference was detected in serum BHB levels between cholesterol high and low-converters from both studies (p = 0.067, paired Wilcoxon signed-rank test), nor positive associations between ketosis and cholesterol-to-coprostanol conversion (Δ BHB vs. Δ coprostanol, R = 0.17, p = 0.39, Spearman’s rank correlation), indicating that the LCHF-induced increase in cholesterol conversion in previous low-converters was not ketosis-dependent.

Thus, LCHF diets consistently increased cholesterol-to-coprostanol conversion in low-converters from the KETO and CARBFUNC cohorts, despite different metabolic health backgrounds and underlying fecal cholesterol and coprostanol concentrations.

### Cholesterol converter type-specific dietary impact on serum lipids

**Figure 6:**
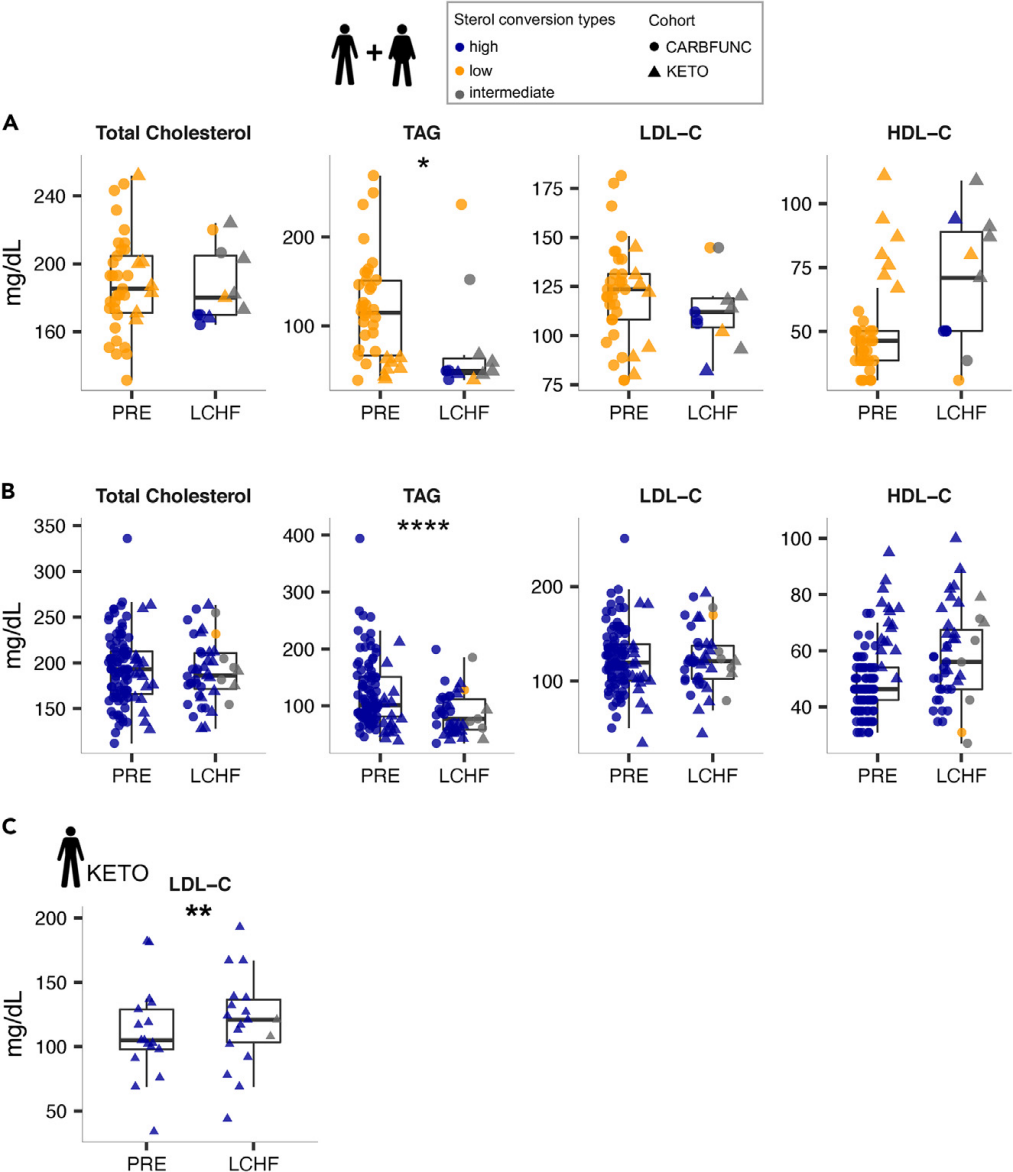
Cholesterol converter type-specific dietary impact on serum lipids (A and B) Decreased serum TAG levels in both cholesterol low-converters (A, N[PRE] = 37, N[LCHF] = 11) and high-converters (B, N[PRE] = 106, N[LCHF] = 43), based on estimated marginal means (EMMs), as determined by the GLMM for the combined KETO and CARBFUNC cohorts. (C) Increased serum LDL-C levels in lean cholesterol high-converters from the KETO study on the LCHF diet (N[PRE] = 17, N[LCHF] = 18). Individuals were classified as cholesterol high/low-converters based on pre-intervention time points, with symbol colors indicating the classification during the LCHF diet. Significance determined by GLMM and post-hoc Tukey’s test (Benjamini-Hochberg-corrected): q > 0.1 ns, q < 0.1 ∗, q < 0.05 ∗∗, q < 0.01 ∗∗∗, q < 0.001 ∗∗∗∗. Pooled data are represented as mean ± SD.

To determine if the cholesterol converter type affected serum lipid responses to LCHF diets, total cholesterol, TAG, HDL-C, and LDL-C concentrations were compared in high and low-converters with and without obesity. The LCHF diet-induced increase in cholesterol conversion in previous low-converters from both cohorts was not accompanied by altered blood lipid concentrations (Figure 6A, q > 0.1, GLMM-based EMMs). However, both cholesterol high and low-converters responded to the LCHF diets with a reduction in serum TAG levels (Figure 6B, q[low] = 0.081, q[high] < 1e-3). This cholesterol converter type-independent effect was apparent even when controlling for cohort-specific differences in serum lipids (q < 0.1, Table S2). However, cholesterol high-converters from the lean KETO cohort responded to the LCHF diet with increased serum LDL-C levels (Figure 6C; Table S2, q = 0.015, GLMM-based EMMs). This effect was not explained by different total fat or saturated, mono-unsaturated, or poly-unsaturated fatty acid intake (E%) between lean cholesterol high and low-converters (Table 1, p > 0.05, Wilcoxon rank-sum test). Lean cholesterol high-converters from the KETO study even consumed less cholesterol during the LCHF diet than low-converters (Table 1, p = 0.016, Wilcoxon rank-sum test). Neither cholesterol high nor low-converters with obesity from the CARBFUNC study exhibited increased serum LDL-C concentrations on the LCHF diet (Table S2, q > 0.05), despite increased saturated fatty acid consumption (30 %E) during the intervention (https://clinicaltrials.gov/ct2/show/NCT03401970), which has previously been suggested to increase LDL-C levels.^38^

In summary, the LCHF diet-induced increase in intestinal cholesterol conversion had no discernible effect on circulating serum lipids but in lean individuals, the cholesterol high-converter type was associated with increased LDL-C levels in response to LCHF diet, independently of fat, cholesterol, and saturated fatty acid intake.

## Discussion

The prospect of reducing cholesterol availability via microbial conversion of endogenous and exogenous cholesterol to non-absorbable coprostanol in the intestine is conceptually appealing as a potential non-pharmacological treatment option for hypercholesterolemia.^9^ However, associations between intestinal and serum cholesterol levels in relation to the gut microbiota, dietary habits, and the metabolic state of the human host, have not been fully resolved. To gain new insights into these relationships and identify robust associations, the present study compared data from two vastly different and geographically independent human cohorts (KETO, CARBFUNC), which are characterized by distinct metabolic backgrounds (individuals with/without obesity), low-carb/high-fat diet interventions (ketogenic/non-ketogenic LCHF diets) and microbiota analysis methods (variable protocols for fecal metagenomic DNA isolation and 16S rRNA gene amplification).

The stratification of healthy human populations into reproducible fractions of high and low cholesterol-to-coprostanol converters has previously been documented for different European countries,^39^^,^^40^ to be stable over the course of several days,^41^ and to shift toward decreased fractions of cholesterol low-converters among older males.^39^^,^^40^ The present study expands on these findings by demonstrating cholesterol conversion to represent an obesity-independent organizational feature of the human fecal microbiome, characterized by conserved proportions of cholesterol high and low-converters, despite adverse metabolic health profiles in CARBFUNC study participants with obesity compared to metabolically healthy lean KETO study participants (increased serum TAG and LDL-C, and decreased HDL-C levels). Cholesterol converting activity has been attributed to a broad and taxonomically diverse group of microbes, including isolates from the genera *Bacteroides*,^42^ *Bifidobacterium*,^43^ *Eubacterium*,^44^ and *Lactobacillus*.^45^ However, most of these bacterial isolates, besides the well-described *E. coprostanoligenes* strain ATCC 51222,^44^^,^^46^^,^^47^ have been lost^2^ and other isolates of the same species failed to show similar activities. Moreover, available genome sequences from these species tested negative for the presence of homologs to the recently identified cholesterol dehydrogenase-encoding *ismA* gene from *E. coprostanoligenes*,^18^ suggesting strain-specific variations in the ability for cholesterol reduction. In this study, in spite of the heterogeneous background of the KETO and CARBFUNC cohort data, cholesterol-to-coprostanol conversion showed a robust positive association with the fecal relative abundance of *E. coprostanoligenes*. Possibly, other bacterial taxa, including *Ruminococcaceae*.UCG.014 and *Lachnoclostridium*, which positively correlated with fecal coprostanol and cholesterol levels, respectively, also contribute to intestinal cholesterol conversion and not all *E. coprostanoligenes* strains may possess the ability to reduce cholesterol. However, the detection of *E. coprostanoligenes* in most individuals (97.6%) and samples (96%) from both cohorts suggests the capacity for cholesterol-to-coprostanol conversion to represent a common and widespread trait of the human gut microbiome, at least in the studied populations. In consequence, cholesterol conversion may be more dependent on intestinal cholesterol availability or diet than on microbiota-related factors, such as the presence of *ismA*-encoding bacteria.^18^

Across the two studied cohorts, intestinal cholesterol-to-coprostanol conversion increased in previous low-converters after transition to LCHF diet, independently of the underlying metabolic background (with/without obesity) or the degree of LCHF diet-induced ketosis. This finding demonstrates the feasibility of modulating intestinal cholesterol conversion via dietary intervention, with potential consequences for intestinal cholesterol availability and absorption. However, there is only limited experimental evidence to support a cholesterol-reducing effect of intestinal cholesterol-to-coprostanol on the host. An inverse relationship between serum cholesterol and the fecal coprostanol/cholesterol ratio was reported in a cohort of 13 hospitalized patients in Japan.^48^ Germ-free rats^49^ and antibiotically treated spontaneously hypercholesterolemic ApoE^−/−^ mice^50^ exhibited elevated serum cholesterol levels. Similarly, fecal microbiota transplantation from humans with excess serum cholesterol to microbiota-depleted ApoE^−/−^ mice produced similar effects,^50^ whereas feeding rabbits with cholesterol-converting *E. coprostanoligenes* reduced circulating cholesterol levels.^51^ However, similar effects could not be induced by feeding *E. coprostanoligenes* to laying hens^52^ or germ-free mice,^47^ despite at least transient intestinal colonization and cholesterol-to-coprostanol activity. Associations of human cholesterol metabolism and gut microbiome, which have previously been reported,^53^ could be mediated by other mechanisms than intestinal cholesterol-to-coprostanol conversion.^9^ For instance, the microbiota is a major determinant of intestinal (primary and secondary, conjugated and unconjugated) bile acid metabolism,^54^ which represents a major route for cholesterol excretion from the body.^55^ Dysbiosis of the gut microbiome could therefore be reflective of a dysregulated human cholesterol or bile acid metabolisms. In the present study, cholesterol high compared to low-converters showed no reduction in circulating cholesterol levels (total cholesterol, LDL-C, HDL-C) and no reduction in serum cholesterol levels was observed in previous low-converters that increased cholesterol conversion in response to LCHF diet. Thus, findings from this study indicate that increasing intestinal cholesterol-to-coprostanol conversion in low-converters with LCHF diet is insufficient as a therapeutic strategy to reduce circulating cholesterol levels.

LCHF diets can induce elevated LDL-C levels in a subset of lean individuals, an observation termed the lean mass hyper-responder (LMHR) phenotype.^56^ The clinical relevance of this phenomenon, which has been controversially debated^30^ and explained with homeostatic adaptations to increased dietary fat intake,^57^ remains incompletely understood. However, the lean mass hyper-responder phenotype received widespread attention on social media and outside of the scientific community,^58^ where LCHF diets continue to be popular among healthy normal-weight individuals.^25^ Therefore, there is a need to identify individuals that are affected by the increased LDL-C response to LCHF diets and to better understand the responsible mechanisms and associated cardiovascular disease risks. In the present study, cholesterol high-converters from the KETO cohort responded to the ketogenic LCHF diet intervention with an increase in serum LDL-C levels. These individuals exhibited increased serum TAG (87.65 mg/dL +/− 47.42, mean ± SD) and decreased HDL-C (69.65 mg/dL+/− 11.46, mean ± SD) before the intervention. While these concentrations fall within the ranges recommended as optimal by the U.S. Centers for Disease Control and Prevention, based on Grundy et al.,^59^ they may indicate a subclinical, adverse metabolic risk profile of the cholesterol high compared to the low-converter types from the lean KETO cohort. In addition, LDL-C levels in lean high-converters on the ketogenic LCHF diet (119 mg/dL +/− 36.05, mean ± SD) stayed below those that were originally described for the lean mass hyper-responder phenotype (LDL-C ≥200 mg/dL^58^). The clinical importance of the observed association between LDL-C and cholesterol conversion should therefore be independently confirmed and individuals with the lean mass hyper-responder LDL-C phenotype tested for cholesterol conversion, in order to assess the diagnostic value of this microbiome trait for personalized response predictions to LCHF diets.

In summary, our findings are in agreement with a model that explains individual intestinal cholesterol-to-coprostanol converter types as a result of long-term dietary or other habits. They do not indicate a hypocholesterolemic effect of intestinal microbial cholesterol conversion but point to an adverse response of increased LDL-C levels to ketogenic diet in lean cholesterol high-converters. Our data suggest potential relevance of the cholesterol converter type as a personalized microbiome marker for metabolic health and response to dietary intervention.

### Limitations of the study

The present study has several limitations: The total numbers of individuals in both cohorts, especially of cholesterol low-converters that represented minor fractions compared to high-converters, were small, providing limited statistical support for comparative analyses among cohort subgroups. In addition, as laid out previously, the two studied cohorts varied with respect to several parameters, including the metabolic background of study participants, the type of diet intervention and the microbiota analysis methods. The diet interventions also involved different durations of six weeks (KETO) and six months (CARBFUNC), although for the latter cohort no difference was detected in fecal sterol and stanol concentrations and serum lipid levels between the three and six-month time points (p > 0.05, Wilcoxon ranked-sum test), attesting to the temporal robustness of the reported findings. Moreover, the detection of shared features between the studied cohorts, such as conserved fractions of cholesterol high/low-converters, shared taxonomic microbiota correlations and diet-induced alterations shifts in cholesterol conversion, demonstrates the strength of the identified associations. It is possible, however, that additional, subgroup-specific associations of the cholesterol converter type with fecal microbiota or serum lipid profiles could not be detected due to the heterogeneous sample collection. The present study relied on 16S rRNA gene amplicon sequence data to describe taxonomic microbiota compositions, without the additional functional, gene-based, insights that shotgun metagenomic sequence data could provide. However, the mechanistic and genetic basis of cholesterol-to-coprostanol conversion remains incompletely understood and previous studies had problems identifying the genetic determinants of cholesterol-to-coprostanol conversion based on the integrative, paired analysis of fecal metagenomics and metabolomics data comparative analysis of homologous protein-coding genes, using instead a newly generated genome sequence of the known cholesterol converter *E. coprostanoligenes* to identify the cholesterol dehydrogenase gene *ismA*.^18^ Although the lack of metagenomic sequence data therefore represents another limitation of this study, the added value for the present analysis may be limited.

## STAR★Methods

### Key resources table

**Table:** 

REAGENT or RESOURCE	SOURCE	IDENTIFIER
**Biological samples**		

Stool samples KETO cohort	This paper	PRJEB51723
Stool samples CARBFUNC cohort	This paper	PRJEB51775
Serum samples KETO cohort	Urbain et al.^32^	N/A
Serum samples CARBFUNC cohort	This paper	N/A

**Chemicals, peptides, and recombinant proteins**		

3-nitrophenylhydrazones	Sigma Aldrich	Cat#N21804; CAS: 636-95-3
RNAlater	Thermo Fisher Scientific	Cat#AM7021M
Lysozyme	Sigma Aldrich	Cat#L6876 ; CAS: 12650-88-3
Mutanolysin	Sigma Aldrich	Cat#SAE0092; CAS: 55466-22-3
Lysostaphin	Sigma Aldrich	Cat#SAE0091; CAS: 9011-93-2
Proteinkinase K	VWR	Cat#97062-238; CAS: 39450-01-6
RNase A	Thermo Fisher Scientific	Cat#EN0531
Lysis buffer	Zymo Reseach	Cat#D7001-1-50
2x Phusion Master Mix	Thermo Fisher Scientific	Cat#F531L

**Critical commercial assays**		

ZR Fecal DNA Miniprep Kit	Zymo Reseach	Cat#D6010
SequalPrep normalization plate kit 96	Illumina	Cat#A1051001
DNA Clean and Concentrator 5 kit	Zymo Reseach	Cat#D4013
TruSeq Nano DNA LT Library Prep kit	Illumina	Cat#20015964
Quick 16S NGS Library Prep Kit	Zymo Reseach	Cat#D6400
MiSeq Reagent Kit v3, 600 cycles	Illumina	Cat#MS-102-3003

**Deposited data**		

16S rRNA amplicon sequencing data of KETO cohort	This paper	PRJEB51723
16S rRNA amplicon sequencing data of CARBFUNC cohort	This paper	PRJEB51775
Serum lipid, glucose and insulin levels of KETO cohort	This paper, see Table S3	N/A
Serum lipid levels, glucose and insulin of CARBFUNC cohort	This paper, see Table S3	N/A
Fecal metabolite levels of KETO cohort	This paper, see Table S3	N/A
Fecal metabolite levels of CARBFUNC cohort	This paper, see Table S3	N/A

**Oligonucleotides**		

Primers for 16S rRNA amplicon sequencing of KETO cohort	This paper, see Table S4	N/A
Primers for 16S rRNA amplicon sequencing of CARBFUNC cohort, V3-V4 of Quick 16S NGS Library Prep Kit	Zymo Research	Cat#D6400

**Software and algorithms**		

cutadapt (v1.10)	Kechin et al.^67^	https://github.com/marcelm/cutadapt/
DADA2	Callahan et al.^72^	https://benjjneb.github.io/dada2/
Prodi 6.4 basis nutritional database software	Nutri-Science GmbH	https://www.nutri-science.de/software/
QIIME 1 (v1.9.1)	Caporaso et al.^68^	http://qiime.org/
QIIME 2 (v2019.7)	Bolyen et al.^69^	https://qiime2.org/
q2-feature-classifier	Bokulich et al.^70^	https://github.com/qiime2/q2-feature-classifier
R (v3.6.1)	packages for analytical part: vegan, biomformat, phyloseq, moments, nortest, lmerTest, emmeans, sjPlot and ComplexHeatmap	https://www.r-project.org/
SILVA database v132	Quast et al.^71^	https://www.arb-silva.de/documentation/release-132/
bowtie2 (v2.2.5)	Langmead and Salzberg^60^	http://bowtie-bio.sourceforge.net/bowtie2/index.shtml
kneaddata (v0.6.1)	Huttenhower lab	https://github.com/biobakery/kneaddata

### Resource availability

#### Lead contact

Further information and requests for resources and reagents should be directed to and will be fulfilled by the lead contact, W. Florian Fricke (w.florian.fricke@uni-hohenheim.de).

#### Materials availability

This study did not generate new unique reagents.

### Experimental model and study participant details

#### Human subjects

The ketogenic diet (KETO) study is a single-arm before-and-after dietary intervention consisting of an isocaloric, *ad libitum*, ketosis-inducing diet with a total daily energy intake of at least 75% fats, 12-20% proteins and 5-10% carbohydrates for 6 weeks (42 days).^32^ It includes 28 healthy, lean adults with a body mass index (BMI) between 19-30 kg/m^2^, who were followed by daily measurements of urinary ketone bodies and 7-day food questionnaires to ensure dietary compliance and continuous ketosis. Additionally, for 12 subjects blood β-Hydroxybutyric acid (BHB) levels were determined after six weeks.^32^^,^^36^ The study was conducted at the University Medical Center Freiburg, Germany, approved by the Ethics Commission of the Albert-Ludwig University of Freiburg (494/14) and registered at the German Clinical Trials (DRKS00009605).

The CARBFUNC study is a 2-year randomized controlled trial involving 145 adults with obesity (BMI > 30 kg/m^2^ and/or waist circumference >102 cm for men and >88 cm for women) that followed a normocaloric diet (males: 2,500 kcal, females: 2,000 kcal) consisting of a low-carb (<=10 E%), high-fat diet (>=75 E%, with 30 E% from saturated fats) (https://clinicaltrials.gov/ct2/show/NCT03401970). CARBFUNC study participants were instructed to follow nutritional recommendations, to consume at least 500 grams of fruits and vegetables per day and generally rely on high-quality food sources. The study was conducted at the University of Bergen, in collaboration with the Haukeland University Hospital in Bergen, Norway. The study was approved by the Regional Committee for Medical and Health Research Ethics (REC West Norway (2017/621/REK vest) and registered at ClinicalTrials.gov (NCT03401970).

All experiments adhered to the regulations of the KETO and CARBFUNC study review boards. All study procedures were performed in compliance with all relevant ethical regulations. Each participant signed informed consent prior to participation. Information on sex and age are available in this paper’s supplemental information in Table S3.

### Method details

#### Sample and dietary record collection

For the KETO study, fecal samples were self-collected by the participants in RNAlater (Thermofisher) on two days before (PRE: days -2 and 0) and at the end of the dietary intervention period (POST: days 40 and 42), stored in a fridge until drop-off and frozen at -80°C at the facility. Venous blood was drawn at visits on days 0 and 42 after overnight fasting. Semi-quantitative 7-day dietary records for the last week before and during the intervention were obtained and energy, macro- and micronutrient intakes were estimated with the Prodi 6.4 basis nutritional database software (Nutri-Science GmbH). For the CARBFUNC study, fecal samples were self-collected before and at 3 and 6 months of the dietary intervention, stored in the freezer and dropped off at the study facility for further storage at -80°C. Blood samples were drawn at the same study visits after overnight fasting. Fecal samples of both cohorts were shipped on dry ice to the final facility and stored at -80°C until processing.

#### Metabolite analysis of blood and feces

Serum concentrations of total cholesterol, triacylglycerides, LDL-C, and HDL-C were determined at the Institute for Clinical Chemistry and Laboratory Medicine of the University Medical Center Freiburg, Germany (KETO study) and at the Department of Medical Biochemistry and Pharmacology, Haukeland University Hospital, Bergen, Norway (CARBFUNC) according to standardized procedures. To track ketosis in CARBFUNC study participants, β-Hydroxybutyric acid (βHB) was measured in fasting plasma samples by gas-chromatography tandem mass spectrometry (GC-MS). Fecal fatty acid, short-chain fatty acid and sterol/stanol concentrations (KETO study) and fecal sterol/stanol concentrations (CARBFUNC study) were determined at the Department of Clinical Chemistry and Laboratory Medicine of the University Hospital Regensburg, Germany. Fecal samples were prepared as previously described.^61^ In brief, up to 2 g of raw feces were homogenized with 2x 2.5 mL 70%-isopropanol, using a gentleMACS™ Dissociator (Miltenyi Biotec GmbH) and the dry weight (DW) determined for a 1 mL aliquot by overnight drying. Fecal homogenates were diluted to 2.0 mg DW/mL and stored at −80°C. Short-chain fatty acids were determined by LC-MS/MS upon derivatization to 3-nitrophenylhydrazones (3NPH)^62^ and other fecal fatty acids by GC-MS.^63^ Fecal sterols and stanols were measured by LC-high resolution MS (LC-MS/HRMS)^61^ for the KETO cohort and by triple quadrupole Gas Chromatography (GC)-MS/MS^41^ for the CARBFUNC cohort. Both methods were validated and run with the same calibration solution resulting in comparable outcomes. For the CARBFUNC cohort, 17 samples did not meet the required concentration of 2.0 mg DW/mL, so they were excluded from the following analyses.

#### Metagenomic DNA extraction from fecal samples

Fecal samples of the KETO study were processed using a previously described combination of enzymatic digestion and mechanical disruption by bead beating.^64^ Briefly, 300μL of the fecal slurry were centrifuged and the pellet dissolved in 800 μL enzyme mix A (5 μL of Lysozyme 10 mg/mL, 13 μL Mutanolysin 11.7 U/μL, 3.2 μL Lysostaphin 1 mg/mL, 778.8 μL 1x PBS) and transferred to a MP lysing matrix B tube (0.1 mm silica spheres, MP Biomedicals). The enzymatic digestion was initiated by incubation at 37°C for 30 minutes. A second enzymatic step was performed by adding 62 μL of enzyme mix B (10 μL Proteinkinase K 20 mg/mL, 50 μL SDS 10%, 2 μL RNase A 10 mg/mL) and incubation at 55°C for 45 minutes. Mechanical lysis was performed by bead beating at 6m/s for 40 seconds (FastPrep-24, MP Biomedicals, Solon).

For the CARBFUNC study, 100-150 mg of the fecal samples were mechanically lysed for 40 seconds at 6m/s in MP lysing matrix B tubes (0.1 mm silica spheres, MP Biomedicals) containing 700μL lysis buffer (Zymo Research). Metagenomic DNA was isolated from lysates using the ZR Fecal DNA Miniprep Kit (Zymo Research) according to the manufacturer’s recommendation. The DNA was eluted in 100 μL RNase-free water and stored at -20°C.

#### 16S rRNA gene amplification and sequencing

For the KETO cohort, the hypervariable V4 region of the 16S rRNA gene was amplified via PCR using Golay-barcoded primers 515F and 806R,^65^ which were modified by adding 0-7 bp-long internal spacers as previously described.^66^ See Table S4 for a list of primer, barcode and spacer sequences. The PCR reaction was comprised of 10 μl of 2x Phusion Master Mix (Thermo Fisher Scientific, Waltham, USA), 2.5 μL of each primer (final concentration 0.4μM), 0.6 μL dimethyl sulfoxide (DMSO), and 4.4 μL template DNA and was carried out at 98°C for 2 min, with 30 cycles at 98°C for 10 s, 52°C for 15s, and 72°C for 10s and final extension at 72°C for 5 min. Equimolar amounts of all PCR products were extracted with the SequalPrep normalization plate kit 96 (Thermo Fisher Scientific), pooled and concentrated with the DNA Clean and Concentrator 5 kit (Zymo Research). Sequencing libraries were prepared using the TruSeq Nano DNA LT Library Prep kit (Illumina) according to the manufacturer's recommendations.

For the CARBFUNC cohort, 16S rRNA gene fragment amplification and barcoding were performed with the Quick 16S NGS Library Prep Kit (Zymo Research). Samples were diluted to an average DNA concentration of 20ng/μL for amplification of the V3-V4 region of the 16S rRNA gene. After barcode addition samples were normalized to 30ng per sample and pooled. Since samples were sequenced on two consecutive sequencing runs, technical replicates and internal microbial standard (mock) communities were added. The final sequencing libraries were prepared according to the MiSeq System Denature and Dilute Libraries Guide (Illumina, San Diego, USA).

All libraries were sequenced on the Illumina MiSeq instrument (MiSeq Reagent Kit v3, 600 cycles, Illumina) at the University of Hohenheim, Stuttgart, Germany.

#### Sequence processing

For the KETO study, raw sequence reads were trimmed with cutadapt v1.10^67^ and barcodes extracted, paired-end reads merged and demultiplexed with QIIME v1.9.1.^68^ Subsequent preprocessing steps were performed with QIIME2 v2019.7,^69^ including open-reference OTU picking based on 97% sequence similarity and classification of representative sequences with the q2-feature-classifier^70^ against the SILVA database v132.^71^ After chimera checking, 59% of sequences were retained. Singletons and sequencing artifacts were filtered if they contained less than 0.005% of total counts.

For the CARBFUNC study, raw sequences were processed with QIIME2 v2019.,^69^ including the DADA2 plugin^72^ for denoising, adapter trimming and chimera checking. A total of 93.8% (1st run) and 93.7% (2nd run) of all reads were retained after chimera checking, which were combined for subsequent analyses. Features of both runs were clustered based on 99% similarity with open-reference picking and representative sequences classified with the q2-feature-classifier^70^ and mapped against the SILVA database v132.^71^ Sequencing artifacts and singletons were filtered if they contained less than 0.0005% of total counts.

For the combined analysis of sequence data from both cohorts, all samples were rarefied to 1,650 sequences. Summaries of the resulting sequence data and their taxonomic classifications are shown in Table S4. A detailed list of all QIIME options, R and UNIX scripts and commands used for sequence processing and data analysis is provided in Table S5.

### Quantification and statistical analysis

Data visualization and statistics were executed using R (v3.6.1) and the packages *vegan, biomformat, phyloseq, moments, nortest, lmerTest, emmeans, sjPlot and ComplexHeatmap*.

#### Calculation of sample means

Fecal samples of the KETO cohort were collected on two consecutive days, both before and after the intervention, to assess short-term intra-individual microbiota changes. Bray-Curtis dissimilarities within individuals were significantly lower between consecutive days than between time points (p< 1e-3, Wilcoxon rank-sum test). Therefore, to minimize the influence of short-term fluctuations, measurements from consecutive days were merged by using mean read counts.

Samples from the CARBFUNC study were sequenced in two separate sequencing runs. To control for batch effects, technical replicates were visually inspected based on PCoA plots of different β-diversity metrics, including weighted/unweighted UniFrac distances, Jaccard index, Euclidean and Manhattan distance. Bray-Curtis dissimilarities were lower between technical replicates from different sequencing runs than between samples collected from the same individual at different time points, and overall microbiota composition was not different between sequencing runs (R=0.009, p=0.29, Analysis of similarities [ANOSIM]), as opposed to between individuals (R= 0.98, p= 0.001, ANOSIM). For all subsequent analyses, the means of the two technical replicates were used.

For fecal and serum lipid profiles from the CARBFUNC study, mean values were calculated from samples collected at 3 and 6 months of the dietary interventions.

#### Statistical analysis

Normal distribution was evaluated using Anderson-Darling and Shapiro-Wilks tests. Non-normally distributed parameters were analyzed by non-parametric tests, such as pairwise Wilcoxon rank-sum test for group comparisons, Spearman’s rank correlation test for correlation analyses, and the chi-squared test for categorical comparisons. Corrections for false discovery rates were performed with the Benjamini-Hochberg (BH) procedure. Unless indicated otherwise, boxplots show medians and corresponding 95% confidence intervals (CI) and significance thresholds with p/q > 0.05 ns, p/q < 0.05 ∗, p/q < 0.01 ∗∗, p/q < 0.001 ∗∗∗. n values represent the number of individuals and are listed in the figure legends for all statistical tests and significance thresholds. Pooled data is represented as mean +/- standard deviation (SD) and listed in the figure legends.

Linear associations between lipid and microbiota compositions before and after intervention were determined with Generalized Linear Mixed Models (GLMMs). First, taxon associations with fecal sterol conversion and blood lipids were assessed across both cohorts before dietary intervention. Only taxa with a relative abundance of >0.1% of all reads from either all high-converter samples combined or all low-converter samples combined, were considered, a pseudocount of 1 added for zero values, and the resulting relative abundances centered log-ratio (clr)-transformed. To control for cohort-specific biases, the KETO or CARBFUNC cohort was included as a random effect in the GLMMs and also evaluated as a fixed effect in multiple linear fixed effects models (Table S2). Additionally, sex was included as a fixed effect to control for gender biases and only results considered, which were not associated with these confounders. To further determine non-linear associations between the sterol converter type and the taxonomic microbiota composition, a random forest (RF) classifier was trained to predict the class of cholesterol-to-coprostanol converter type (high/low) using clr-transformed relative taxa abundances. The model performance was evaluated based on different numbers of trees (250, 500, 1000 and 2000), as well as different numbers of variables tried at each split (mtry) with values ranging from 1 to 20. The final RF classifier was run with 501 trees, 12 variables tried at each split, and a default node-size. Leave-one-out cross-validation (LOOCV) was used to evaluate the model accuracy based on recall and precision and to identify the most important features based on the mean decrease in accuracy.

Alterations in the relative abundance of microbial taxa due to the LCHF diets across both cohorts were determined in another GLMM, which was controlled for cohort and sex-specific effects as fixed effects, and repeated measurements per individuum as a random effect. Relative abundances of taxa were clr--transformed after pseudocount addition similar as described above. Microbial taxa with a relative abundance above 0.5% and which are detected in at least 4 samples, with a fold change < -0.25 or > 0.25 as determined based on estimated marginal means (EMMs) before and after the intervention, and significance of q < 0.1 based on FDR-corrected Tukey’s test, for which no cohort or gender-bias was detected, were considered (Table S2).

Changes in the relative abundance of sterol conversion associated microbial taxa, as well as in fecal sterols and serum lipids, in response to both, the dietary intervention and the sterol conversion type, were evaluated in GLMMs with dietary intervention and the pre-existing sterol conversion type as interactive fixed effects. Similar to previous models, cohort and sex-associations were controlled for as fixed effects as well repeated measurements as random effects per individuum. Relative abundances of taxa were clr-transformed after pseudocount addition similar as described above . For serum lipids and fecal sterols, different models (Gaussian and Gamma distribution) and link functions (identity, log, inverse) were compared based on Akaike information criterion (AIC) and diagnostic plots, and the best fit was determined for a log-transformed Gaussian distribution. To achieve normal distribution and improve skewness and kurtosis, pseudocounts of 0.1 instead of zero values were added for serum and fecal metabolites and concentrations log-transformed (Table S2). Differences between subgroups were further assessed by calculation of EMMs and post hoc comparisons by Tukey’s test. As serum lipid levels were associated with cohorts in these models, the same interaction GLMM was also applied for each cohort separately with the exclusion of cohort as fixed effect (Table S2).

For all GLMMs, fits were assessed using diagnostic plots and for model and EMMs comparisons, significance was determined after BH-based false discovery rate correction (FDR) with the following thresholds for q-values: q>0.1 ns, q<0.1 ∗, q<0.05 ∗∗, q<0.01 ∗∗∗ and q<0.001 ∗∗∗∗. Models with indications for a problematic fit, e.g. with a singularity warning, were not considered. Odds ratios with 95% CI, as well as marginal and conditional R^2^ were calculated for all significant models (Table S2).

### Additional resources

The KETO study was registered at the German Clinical Trials (DRKS00009605). Further information on the study protocol, intervention arms and inclusion criteria are available under: https://drks.de/search/de/trial/DRKS00009605.

The CARBFUNC study was registered at ClinicalTrials.gov (NCT03401970). Further information on the study protocol, intervention arms and inclusion criteria are available under: https://classic.clinicaltrials.gov/ct2/show/NCT03401970.

## Data Availability

Sequence data from 16S rRNA gene amplicon sequencing-based taxonomic fecal microbiota analysis have been deposited at the European Nucleotide Archive (ENA) at EMBL-EBI (ENA: PRJEB51723 (KETO), PRJEB51775 (CARBFUNC)) and are publicly available. Before submission, sequences were mapped against the human genome hg38 via bowtie2^60^ implemented in kneaddata workflow v0.6.1 [https://github.com/biobakery/kneaddata] and host sequences were removed. Fecal and serum metabolite compositions are provided in Table S3 as well as taxa count tables in Tables S4. A list of all tools and commands used for sequence preprocessing and analyses including original R code is available in this paper’s supplemental information in Table S5. Any additional information required to reanalyze the data reported in this paper is available from the lead contact upon request.

## Supplemental information

Document S1. Figures S1–S3 and Table S1

Table S2. Associations between taxonomic microbiota compositions, serum lipids and cholesterol-to-coprostanol conversion as determined by generalized linear mixed effects models, related to Figures 2, 5, 6, and S1

Table S3. Metadata for the KETO and CARBFUNC cohorts

Table S4. Fecal microbiota genus-level taxa counts

Table S5. QIIME options, R and UNIX scripts and commands used for sequence processing and data analysis
