# Supplementary material for: High-fat diet impact on intestinal cholesterol conversion by the microbiota and serum cholesterol levels
Source: iScience. 2023 Aug 21;26(9):107697. doi: 10.1016/j.isci.2023.107697 (PMC10485154; doi:10.1016/j.isci.2023.107697)
Supplement: Document S1. Figures S1–S3 and Table S1 [file mmc1.pdf]

iScience, Volume *26*

Supplemental information

High-fat diet impact on intestinal cholesterol conversion by the microbiota and serum cholesterol levels

Alena M. Bubeck, Paul Urbain, Cathrine Horn, Anna S. Jung, Lisa Ferrari, Hannah K. Ruple, Daniel Podlesny, Stefanie Zorn, Johnny Laupsa-Borge, Caroline Jensen, Inge Lindseth, Gülen Arslan Lied, Jutta Dierkes, Gunnar Mellgren, Hartmut Bertz, Silke Matysik, Sabrina Krautbauer, Gerhard Liebisch, Hans-Frieder Schoett, Simon N. Dankel, and W. Florian Fricke

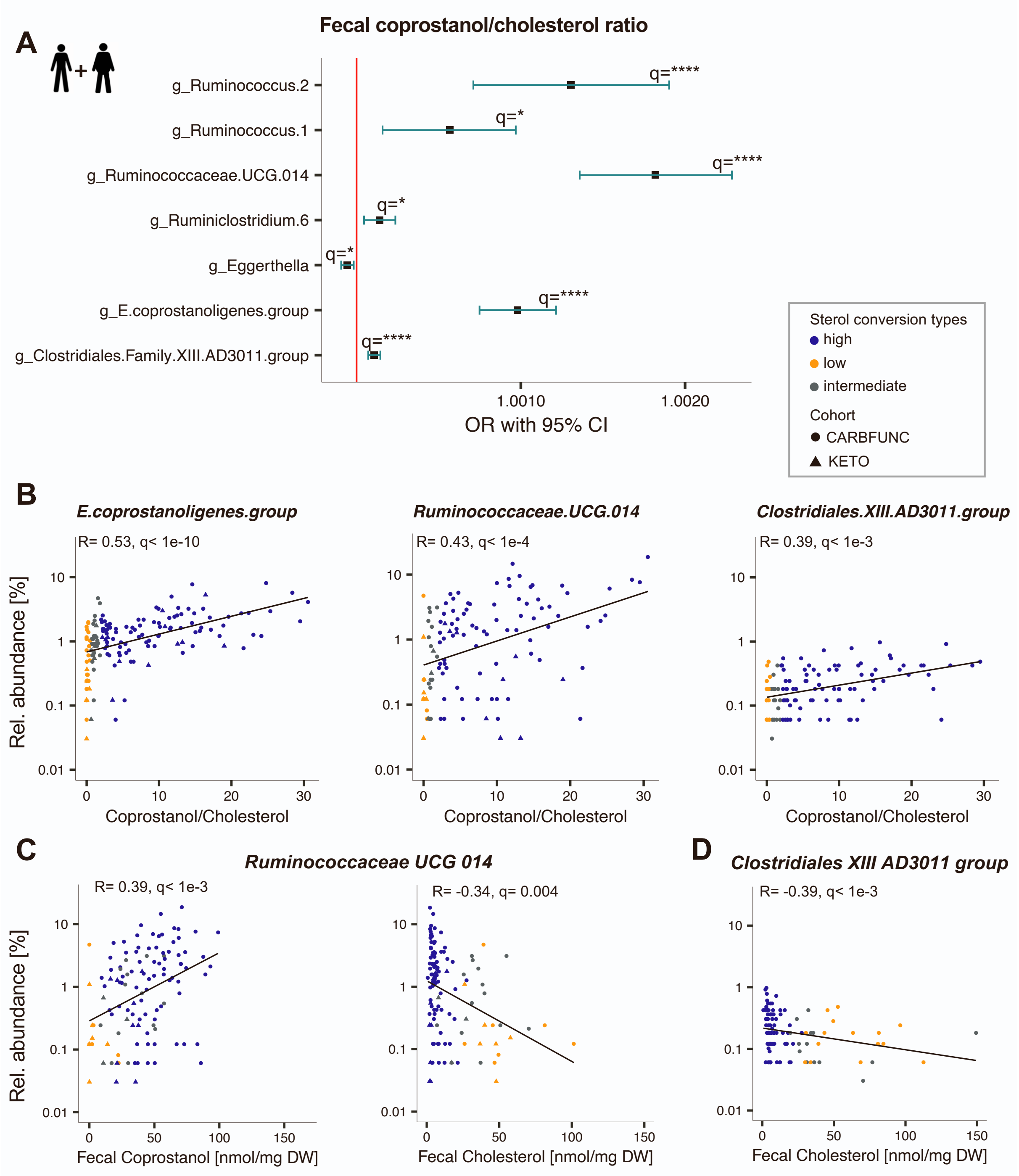

Figure S1. Associations of fecal taxonomic microbiota composition with the fecal coprostanol/cholesterol ratio, Related to Figure 2.

(A) Positive and negative associations of bacterial taxa with the fecal coprostanol/cholesterol ratio, as identified by the generalized linear mixed model (GLMM) for the combined dataset of KETO (n = 23) and CARBFUNC (n = 145) study participants. For the GLMM input, zero values were replaced with a pseudocount and cohort and gender added as random and fixed effects (see Methods for details).

(B) Significant associations with the fecal coprostanol/cholesterol ratio, as supported by Spearman's rank correlation analysis, of *Eubacterium coprostanoligenes*.group (n = 158), *Ruminococcaceae*.UCG.014 (n = 95) and *Clostridiales*.XIII.AD3011 (n = 111, only detected in CARBFUNC samples).

(C) Across both cohorts combined, *Ruminococcaceae*.UCG.014 (n = 95) was positively correlated with fecal coprostanol and negatively correlated with fecal cholesterol concentrations.

(D) *Clostridiales*.XIII.AD3011 (n = 111) was only detected in CARBFUNC samples and negatively correlated with fecal cholesterol concentrations.

For all analyses, p-values were Benjamini-Hochberg (BH) corrected: q > 0.1 ns, q < 0.1 *, q < 0.05 **, q < 0.01 ***, q < 0.001 ***

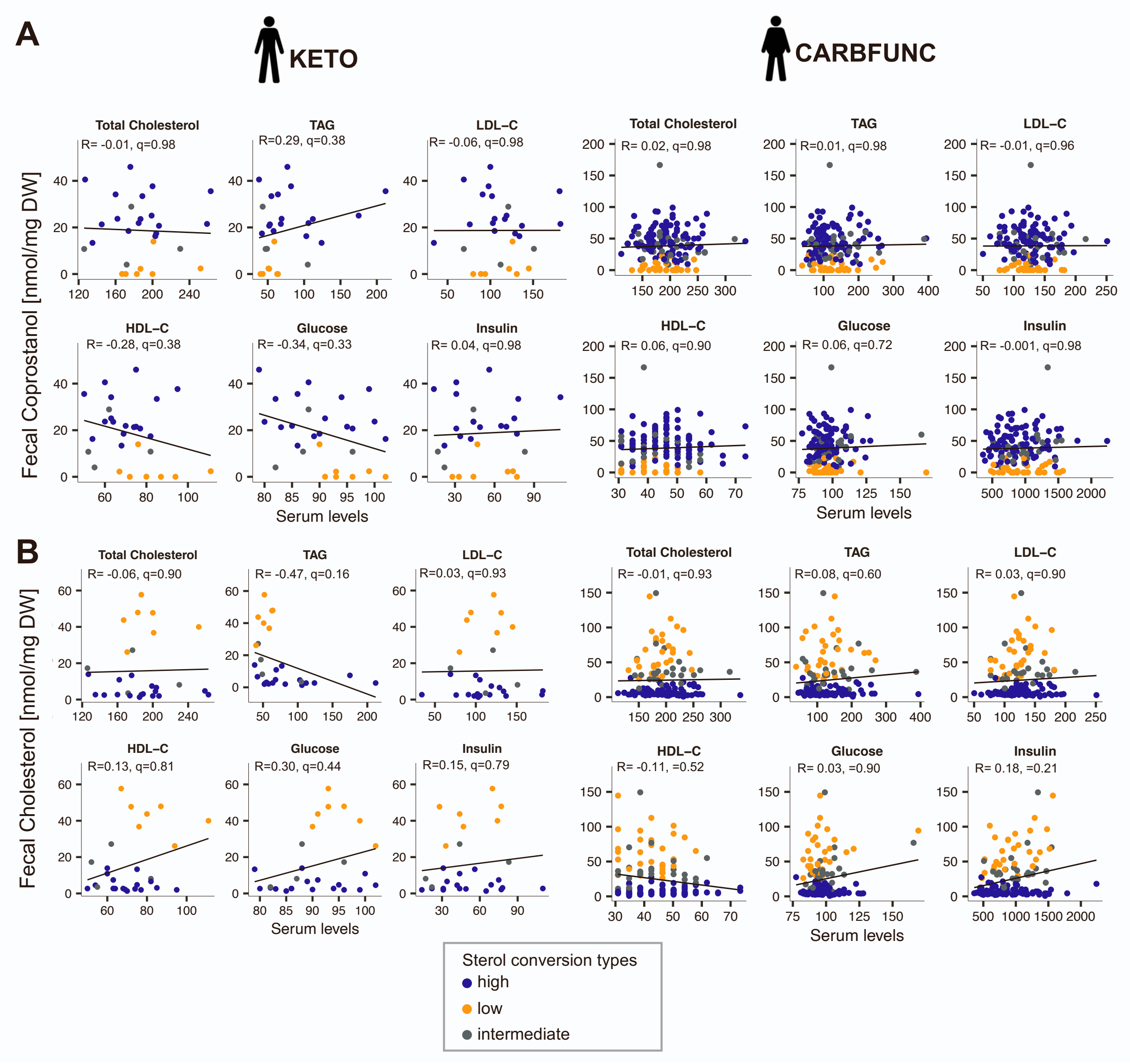

Figure S2. No associations between fecal cholesterol and coprostanol concentrations and serum lipid levels in both cohorts, Related to Figure 4. Neither fecal cholesterol nor coprostanol levels were significantly correlated with serum lipids in the KETO or CARBFUNC cohorts. All serum lipids and blood glucose were measured as mg/dL and insulin levels as pmol/L. Spearman's rank correlation with Benjamini-Hochberg (BH) correction. $n_{KETO} = 28$, $n_{CARBFUNC} = 145$; Abbreviations: TAG, triacylglycerides; LDL-C, low-density lipoprotein cholesterol; HDL-C, high-density lipoprotein cholesterol.

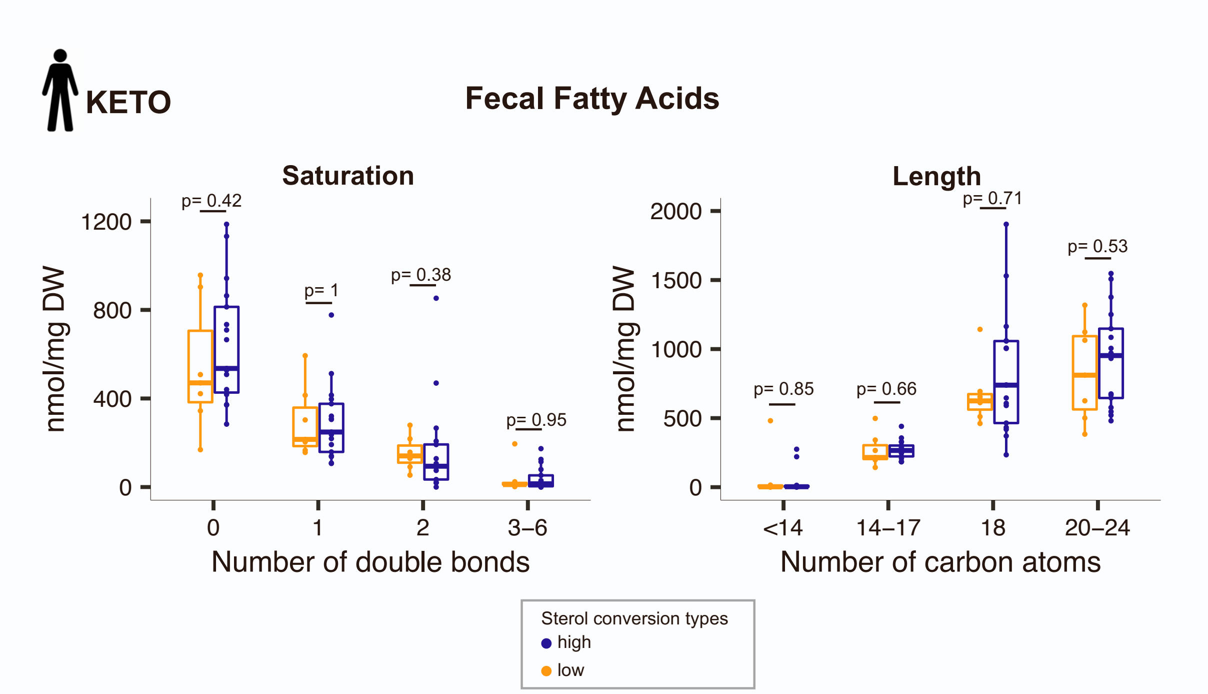

Figure S3. Similar fecal fatty acid profiles in cholesterol high and low-converters from the KETO cohort before the LCHF diet intervention, Related to Figure 5. Wilcoxon rank-sum test, $p > 0.05$ ns, n(low-converter) = 7, n (high-converter) = 17. Data are represented as mean +/- SD.

Table S1. Overview of KETO and CARBFUNC study cohort parameters, Related to Figures 1 and 4. Table shows mean values +/- standard deviations (sd). Continuous variables were compared with the Wilcoxon rank-sum test, categorical variables with the Chi-Square test. p > 0.05 ns, p < 0.05 *, p < 0.01 **, p < 0.001 ***; Abbreviations: TAG, triacylglycerides; LDL-C, low-density lipoprotein cholesterol; HDL-C, high-density lipoprotein cholesterol; DW, dry weight.

	KETO (n=28) mean ± sd	CARBFUNC (n=145) mean ± sd	p	Significance thresholds
Sex (female vs male)	20 vs 8	72 vs 73	0.041	*
Age [years]	37 ± 11.73	43 ± 8.32	0.023	***
BMI	23.89 ± 3.14	36.86 ± 4.62	< 1e-22	***
Weight [kg]	70.23 ± 11.51	112.16 ± 18.05	< 1e-20	***
Serum metabolites [mg/dL]				
Total cholesterol	186.48 ± 34.73	193.91 ± 36.64	0.32	ns
TAG	78.57 ± 39.08	130.46 ± 60.93	< 1e-7	***
HDL-C	71.26 ± 14.29	44.80 ± 9.07	< 1e-13	***
LDL-C	110.86 ± 31.33	126.74 ± 31.6	0.03	*
TAG/HDL-C	1.13 ± 0.82	3.19 ± 2.03	< 1e-11	***
LDL-C/HDL-C	1.60 ± 0.60	2.98 ± 1.07	< 1e-10	***
Glucose	90.79 ± 6.60	97.54 ± 12.67	0.002	***
Insulin [pmol/L]	50.39 ± 23.91	921.51 ± 332.29	< 1e-16	***
Fecal metabolites [nmol/mg DW]				
Coprostanol	18.71 ± 13.44	38.75 ± 27.05	< 1e-3	***
Cholesterol	15.76 ± 17.54	24.46 ± 29.13	0.054	ns
Coprostanol/Cholesterol	5.50 ± 5.49	6.38 ± 7.31	0.62	ns
Sitosterol	4.62 ± 3.99	6.21 ± 6.89	0.64	ns
Stigmastanol	10.39 ± 6.71	9.75 ± 6.44	0.54	ns
Campesterol	1.43 ± 1.51	2.49 ± 3.33	0.07	ns
Acetate	223.09 ± 183.82	158.35 ± 110.33	0.086	ns
Propionate	82.47 ± 67.7	62.64 ± 47.37	0.19	ns
Butyrate	62.85 ± 49.25	58.2 ± 51.68	0.57	ns
Iso-Butyrate	10.3 ± 6.65	8.19 ± 6.59	0.015	*
